# The complete chloroplast genome sequence of *Olea ferruginea*

**DOI:** 10.1080/23802359.2019.1664948

**Published:** 2019-09-12

**Authors:** Yi Wang, Jiabo Hao, Xiaolong Yuan, Bin Lu

**Affiliations:** Laboratory of Forest Plant Cultivation and Utilization, Yunnan Academy of Forestry, Kunming, Yunnan, People’s Republic of China

**Keywords:** *Olea ferruginea*, chloroplast, Illumina sequencing, phylogenetic analysis

## Abstract

The first complete chloroplast genome sequences of *Olea ferruginea* were reported in this study. The cpDNA of *O. ferruginea* is 155,531 bp in length, contains a large single-copy region (LSC) of 86,279 bp and a small single-copy region (SSC) of 17,790 bp, which were separated by a pair of inverted repeat (IR) regions of 25,731 bp. The genome contains 129 genes, including 84 protein-coding genes, 8 ribosomal RNA genes, and 37 transfer RNA genes. The overall GC content of the whole genome is 37.8%. Phylogenetic analysis of six chloroplast genomes within the genus *Olea* suggests that *O. ferruginea* is closely related to *Olea europaea* subsp. *cuspidate*.

*Olea ferruginea* Royle is the species of the genus *Olea* within the family Oleaceae, and whose natural distribution in Asia (Lumaret et al. 2000). It is an important multipurpose tree and an underutilized fruit tree crop (Sharma et al. 2013). *Olea ferruginea* is one of the most important plants in folk medicine in Pakistan, their leaves showed good inhibition against TNALP and CIALP enzymes as well as a very good anticancer activity on HeLa cell-line (Hashmi et al. 2015). The ripe fruits are also a rich source of natural antioxidants (Anwar et al. 2013). However, there has been no genomic studies on *O. ferruginea*.

Herein, we reported and characterized the complete *O. ferruginea* plastid genome (MN017130). One *O. ferruginea* individual (specimen number: 2018100012) was collected from Kunming botanical garden, Kunming, Yunnan Province of China (25°14'16'' N, 102°75'13'' E). The specimen is stored at Yunnan Academy of Forestry Herbarium, Kunming, China and the accession number is YAFH0012982. DNA was extracted from its fresh leaves using DNA Plantzol Reagent (Invitrogen, Carlsbad, CA).

Paired-end reads were sequenced by using Illumina HiSeq system (Illumina, San Diego, CA). In total, about 22.2 million high-quality clean reads were generated with adaptors trimmed. Aligning, assembly, and annotation were conducted by CLC de novo assembler (CLC Bio, Aarhus, Denmark), BLAST, GeSeq (Tillich et al. 2017), and GENEIOUS v 11.0.5 (Biomatters Ltd, Auckland, New Zealand). To confirm the phylogenetic position of *O. ferruginea*, other five species of genus *Olea* from NCBI were aligned using MAFFT v.7 (Katoh and Standley 2013) and maximum-likelihood (ML) bootstrap analysis was conducted using RAxML (Stamatakis 2006); bootstrap probability values were calculated from 1000 replicates. *Syringa yunnanensis* (MH817943) and *Syringa vulgaris* (MG255768) were served as the out-group.

The complete *O. ferruginea* plastid genome is a circular DNA molecule with the length of 155,531 bp, with a large single copy (LSC: 86,279 bp), a small single copy (SSC: 17,790 bp), and two inverted repeats (IRa and IRb: 25,731 bp each). The overall GC content of the whole genome is 37.8%, and the corresponding values of the LSC, SSC, and IR regions are 35.8, 31.9, and 43.2%, respectively. The genome contains 129 genes, including 84 protein-coding genes, 8 ribosomal RNA genes, and 37 transfer RNA genes. Phylogenetic analysis showed that *O. ferruginea* clustered together with *Olea europaea* subsp. *cuspidate*, which indicated the phylogenesis classification of *O. ferruginea* (Figure 1). The determination of the complete plastid genome sequences provided new molecular data to illuminate the *Olea* evolution.

**Figure 1.**
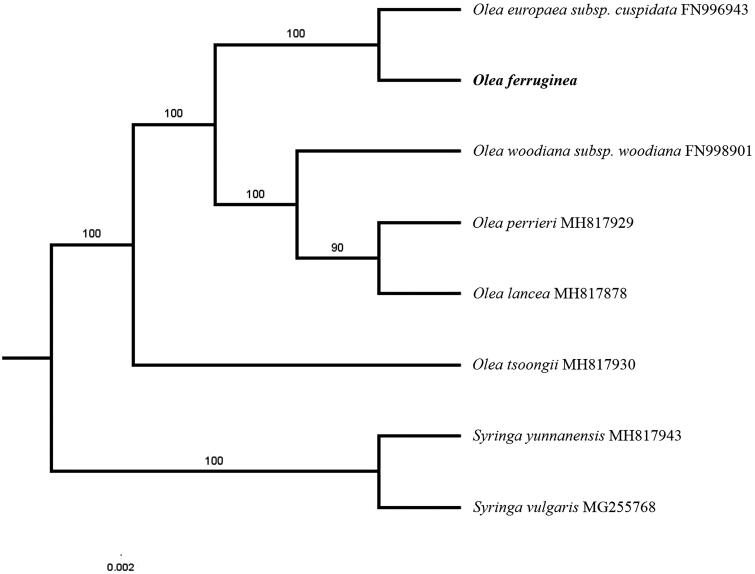
The maximum-likelihood tree based on the 6 chloroplast genomes of genus Olea. The bootstrap value based on 1000 replicates is shown on each node.
